# Inhibition of Lipopolysaccharide-Induced Inflammatory and Oxidative Responses by *Trans*-cinnamaldehyde in C2C12 Myoblasts

**DOI:** 10.7150/ijms.59169

**Published:** 2021-04-23

**Authors:** Cheol Park, Hyesook Lee, Suhyun Hong, Ilandarage Menu Neelaka Molagoda, Jin-Woo Jeong, Cheng-Yun Jin, Gi-Young Kim, Sung Hyun Choi, Sang Hoon Hong, Yung Hyun Choi

**Affiliations:** 1 Division of Basic Sciences, College of Liberal Studies, Dong-Eui University, Busan 47340, Republic of Korea.; 2 Anti-Aging Research Center, Dong-eui University, Busan 47340, Republic of Korea.; 3 Department of Biochemistry, Dong-eui University College of Korean Medicine, Busan 47227, Republic of Korea.; 4 Department of Marine Life Sciences, School of Marine Biomedical Sciences, Jeju National University, Jeju 63243, Republic of Korea.; 5 Nakdonggang National Institute of Biological Resources, Sangju 37242, Republic of Korea.; 6 School of Pharmaceutical Sciences, Zhengzhou University, Henan 450001, China.; 7 Department of System Management, Korea Lift College, Geochang 50141, Republic of Korea.; 8 Department of Internal Medicine, Dong-eui University College of Korean Medicine, Busan 47227, Republic of Korea.

**Keywords:** *Trans*-cinnamaldehyde, inflammation, oxidative stress, TLR4/NF-κB, Nrf2/HO-1.

## Abstract

**Background:** *Trans*-cinnamaldehyde (tCA), a bioactive component found in *Cinnamomum cassia*, has been reported to exhibit anti-inflammatory and antioxidant effects, but its efficacy in muscle cells has yet to be found. In this study, we investigated the inhibitory effect of tCA on inflammatory and oxidative stress induced by lipopolysaccharide (LPS) in C2C12 mouse skeletal myoblasts.

**Methods:** To investigate the anti-inflammatory and antioxidant effects of tCA in LPS-treated C2C12 cells, we measured the levels of pro-inflammatory mediator, cytokines, and reactive oxygen species (ROS). To elucidate the mechanism underlying the effect of tCA, the expression of genes involved in the expression of inflammatory and oxidative regulators was also investigated. We further evaluated the anti-inflammatory and antioxidant efficacy of tCA against LPS in the zebrafish model.

**Results:** tCA significantly inhibited the LPS-induced release of pro-inflammatory mediators and cytokines, which was associated with decreased expression of their regulatory genes. tCA also suppressed the expression of Toll-like receptor 4 (TLR4) and myeloid differentiation factor, and attenuated the nuclear translocation of nuclear factor-kappa B (NF-κB) and the binding of LPS to TLR4 on the cell surface in LPS-treated C2C12 cells. Furthermore, tCA abolished LPS-induced generation of ROS and expression levels of ROS producing enzymes, NADPH oxidase 1 (NOX1) and NOX2. However, tCA enhanced the activation of nuclear translocation of nuclear factor-E2-related factor 2 (Nrf2) and the expression of heme oxygenase-1 (HO-1) in LPS-stimulated C2C12 myoblasts. In addition, tCA showed strong protective effects against NO and ROS production in LPS-injected zebrafish larvae.

**Conclusions:** Our findings suggest that tCA exerts its inhibitory ability against LPS-induced inflammatory and antioxidant stress in C2C12 myoblasts by targeting the TLR4/NF-κB, which might be mediated by the NOXs and Nrf2/HO-1 pathways.

## Introduction

As the largest tissue and one of the most active metabolic organs in the body, skeletal muscles play a key role in supporting body structures, controlling movement and storing energy. Skeletal muscle has also been recognized as one of the endocrine organs that produce and release cytokines, metabolites, and hormones that are important for maintaining human homeostasis 1,2. In addition, because skeletal muscle expresses abundant toll-like receptor (TLR) 4, it is sensitive to circulating endotoxins such as lipopolysaccharide (LPS), a component of the extracellular membrane of Gram-negative bacteria 3,4. In muscle cells stimulated by circulating LPS, the expression of inflammatory cascade effector enzymes and cytokines is increased by activation of the TLR4-mediated intracellular signaling pathways, including nuclear factor-kappa B (NF-κB) 4,5. Nitric oxide (NO) and prostaglandin E_2_ (PGE_2_) are representative pro-inflammatory mediators, and pro-inflammatory cytokines such as tumor necrosis factor-alpha (TNF-α), interleukin (IL)-6, IL-1β, and interferon-γ facilitate inflammation 6,7. Moreover, the expression of inducible NO synthase (iNOS) and cyclooxygenase-2 (COX-2), which are involved in the production of NO and PGE_2_, respectively, is positively correlated with the expression of pro-inflammatory cytokines 8,9.

On the other hand, reactive oxygen species (ROS) and related species, which are required for proper physiological function under normal conditions, serve as important signaling molecules that are closely related to host defense responses. However, oxidative stress, characterized by the excessive production of ROS, contributes to the progression of the inflammatory response and multiple diseases 10,11. LPS can induce and accelerate oxidative stress along with the inflammatory cascade. In this respect, accumulated evidences established that LPS-induced inflammation and oxidative stress is closely related to several pathological conditions including endometritis, periodontitis and mastitis 12-14. Upon the LPS stimulation of muscle cells, the production of ROS is increased, contributing to the manifestation of inflammation, and overproduced inflammatory factors may promote excessive ROS production 4,15. Currently, non-steroidal anti-inflammatory drugs are widely used to suppress inflammatory symptoms and relieve oxidative stress, but various side effects have been reported from long-term use 16,17. Therefore, research on reliable and effective alternative agents for the prevention and treatment of various diseases is urgently required.

*Trans*-cinnamaldehyde (tCA) is one of the major phytochemical constituents isolated from the stem bark of *Cinnamomum cassia* (L.) J. Presl (Cinnamon), which is frequently used in traditional medicine for the prevention and treatment of various diseases such as ischemia, anxiety, arrhythmia, indigestion, diabetes, gastritis, blood circulation disorders, and so on 18-20. A number of previous studies have shown that tCA has a variety of beneficial pharmacological effects, including anti-inflammatory, antioxidant, anti-diabetic, anti-obesity, neuroprotective, and anti-tumor effects in a number of *in vitro* and *in vivo* models 21-27. As an example of the anti-inflammatory efficacy of tCA, Wang et al. 28 recently reported that this compound has a beneficial effect on inflammation-mediated depression, and possible mechanisms may include blocking the NF-κB pathway. Xia et al. 29 also suggested that the anti-inflammatory of tCA could be mediated by modulating the NF-κB signaling pathway in an osteoarthritis model. Additionally, tCA has been reported to inhibit the inflammatory response in LPS-stimulated monocytes and microglia by suppressing the activity of NF-κB 30,31. These previous studies suggest that inhibition of the NF-κB signaling pathway plays a central role in the anti-inflammatory efficacy of tCA. Moreover, the exogenous addition of tCA has been shown to induce the expression of nuclear factor-E2-related factor 2 (Nrf2) and heme oxygenase-1 (HO-1), thereby increasing the effect of treadmill exercise on learning and memory in cognitive impaired mice 32. These results well support the previous studies that Nrf2 plays a key role in the protective effect of tCA against oxidative stress-induced lung fibroblast and hippocampus injury 33,34 suggesting that tCA may have strong potential in the prevention or treatment of diseases associated with inflammatory and oxidative stress. However, the correlation between the anti-inflammatory and antioxidant effects of tCA in muscle cells has not been investigated yet. Therefore, in this study, we investigated the inhibitory effect of tCA on the inflammatory and oxidative reactions induced by LPS using C2C12 mouse skeletal muscle cells. We also demonstrated the anti-inflammatory and antioxidant potential of tCA in the zebrafish larvae model.

## Materials and Methods

### Cell culture

The C2C12 cell line, an immortalized mouse myoblast cell line, was purchased from the American Type Culture Collection (Manassas, VA, USA). The cells were maintained in humidified air at 37°C, and 5% CO_2_ in Dulbecco's modified Eagle's medium (DMEM) containing 100 U/ml penicillin and streptomycin, and 10% fetal bovine serum. All materials required for the cell culture were purchased from WelGENE Inc. (Daegu, Republic of Korea). tCA and LPS were purchased from Sigma-Aldrich Chemical Co. (St. Louis, MO, USA). They were dissolved in dimethyl sulfoxide (DMSO, Sigma-Aldrich Chemical Co.) and distilled water to make the stock solutions, respectively. Each stock solution (tCA 1 mM, LPS 100 mg/ml) was appropriately diluted in the complete culture medium and used to treat C2C12 cells.

### Cell viability assay

The cytotoxicity of tCA against C2C12 cells in the presence or absence of LPS was determined using the 3-(4,5-dimethylthiazol-2-yl)-2,5-diphenyltetrazolium bromide (MTT) reduction assay. In brief, the cells were seeded into 96-well culture plates at a density of 1×10^4^ cells/ml. After cultivation for 24 h, cells were treated with various concentrations of tCA alone or pre-treated with the indicated concentrations of tCA for 1 h before 1 mg/ml LPS treatment for 24 h. Then, the medium was removed, and MTT solution (0.5 mg/ml, Sigma-Aldrich Chemical Co.) was dispensed into each well and reacted at 37°C as previously described 35. After 3 h, the supernatant was removed and DMSO was added to dissolve the blue formazan crystals for 10 min. The absorbance per well was quantified at a wavelength of 540 nm using an enzyme-linked immunosorbent assay (ELISA) plate reader (Dynatech Laboratories, Chantilly, VA, USA).

### Measurement of NO, PGE_2_, and cytokines

The cells were incubated in 24-well culture plates at a density of 5×10^4^ cells/ml for 24 h, followed by treated with 10 mM or 20 mM tCA for 1 h and then stimulated with 1 mg/ml LPS for 24 h. The NO level in the medium was evaluated by the amount of nitrite measured using the Griess reagent (Sigma-Aldrich Chemical Co.) as previously described 36. Briefly, 100 μL of the cell-conditioned medium was mixed with the same amount of Griess reagent for 10 min. The absorbance was measured at 540 nm using an ELISA reader and calculated by comparison to a sodium nitrite (NaNO_2_) standard curve. To investigate the PGE_2_ and cytokine levels, the culture supernatants were collected and assayed using commercially available ELISA kits (R&D Systems Inc., Minneapolis, MN, USA) according to the instructions from the manufacturer. The absorbance was measured at a wavelength of 450 nm using an ELISA reader as previously described 36.

### Reverse transcription-polymerase chain reaction (RT-PCR) assay

The cells were incubated in 6-well culture plates at a density of 3×10^5^ cells/ml for 24 h, followed by treated with 10 mM or 20 mM tCA for 1 h and then stimulated with 1 mg/ml LPS for 24 h. Total RNA was isolated from the cells using TRIzol reagent (Invitrogen Life Technologies, Carlsbad, CA, USA), following the manufacturer's instructions, and quantified. The isolated total RNA (1 μg) was used to synthesize cDNA using AccuPower^®^ RT PreMix (Bioneer, Daejeon, Republic of Korea) according to the manufacturer's instructions. The cDNA generated at room temperature (RT) was amplified using the One-Step RT-PCR PreMix Kit with selected primers (iNtRON Biotechnology Inc., Seongnam, Republic of Korea). Information on the primers used is provided in Table 1. The amplified DNA products were electrophoresed on 1.5% agarose gels and visualized after ethidium bromide (EtBr, Sigma-Aldrich Chemical Co.) staining as previously described 37. Densitometric analysis of the bands was performed using the ImageJ® software (v1.48, NIH, Bethesda, MD).

### Protein isolation and Western blot analysis

The cells were incubated in 100 mm culture dish at a density of 2×10^6^ cells/ml for 24 h, followed by treated with 10 mM or 20 mM tCA for 1 h and then stimulated with 1 mg/ml LPS for 24 h. To extract proteins, the cells were washed with cold phosphate-buffered saline (PBS) and lysed with lysis buffer as previously described 38. In parallel, nuclear extraction reagents (Thermo Fisher Scientific, Waltham, MA, USA) were used to isolate proteins from the nucleus and cytoplasm according to the manufacturer's protocol. The concentration of the isolated protein was measured using the Bio-Rad protein assay kit obtained from Bio-Rad Laboratories (Hercules, CA, USA). Equal amounts of protein were separated by 8% ~ 15% sodium dodecyl sulfate-polyacrylamide gel electrophoresis. Proteins in the gel were subsequently transferred to polyvinylidene difluoride membranes (Schleicher and Schuell GmbH, Keene, NH, USA). The protein-transferred membranes were blocked with non-fat dry milk solution (5%) at RT for 1 h, and then reacted with primary anti-bodies (1: 1,000) obtained from Santa Cruz Biotechnology, Inc. (Santa Cruz, CA, USA) and Cell Signaling Technology (Beverly, MA, USA) overnight at 4°C. The membranes were washed three times for 5 min with Tris-buffered saline (0.1% Tween-20) and then incubated with goat anti-rabbit IgG-horseradish-peroxidase (HRP) and goat anti-mouse IgG-HRP secondary antibodies (1:10,000; Santa Cruz Biotechnology, Inc.) for 2 h at RT. The membrane was reacted with an enhanced chemiluminescent solution purchased from Amersham Corp. (Arlington Heights, IL, USA) and then exposed to X-ray film to visualize the corresponding proteins. Densitometric analysis of the bands was performed using the ImageJ® software (v1.48, NIH, Bethesda, MD).

### Immunofluorescence for NF-κB

C2C12 cells were seeded into 4-well cell culture slides at a density of 5×10^4^ cells/ml and stabilized for 24 h. The cells were pre-treated with 20 mM tCA for 1 h and then treated with or without 1 mg/ml LPS for 1 h. After treatment, the cells were fixed with ice-cold methanol for 10 min and washed with PBS. Subsequently, the cells were blocked using 5% bovine serum albumin (BSA, Sigma-Aldrich Chemical Co.) with PBS-T (PBS containing 0.1% Triton X) for 1 h and then incubated with anti-NF-κB p65 (1:100 in 2.5% BSA in PBS-T) at 4°C overnight. The cells were washed with PBS-T and incubated with the secondary antibody (goat anti-rabbit IgG cross-absorbed secondary antibody conjugated to Alexa Fluor (AF) 594, Thermo Fisher Scientific) for 1 h. After washing with PBS, the cells were counterstained with 4',6-diamidino-2-phenylindole (DAPI, Sigma-Aldrich Chemical Co.) for 20 min. Cell fluorescence was observed using a fluorescence microscope (Carl Zeiss, Oberkochen, Germany) at Core-Facility Center for Tissue Regeneration (Dong-eui University, (Busan, Republic of Korea).

### Measurement of LPS binding levels on cell surface

C2C12 cells were seeded into 4-well cell culture slides at a density of 5×10^4^ cells/ml and stabilized for 24 h. To explore the inhibitory effect of tCA on TLR4 expression on the cell surface of C2C12 cells, the cells pre-treated with 20 μM tCA for 1 h were further treated with 1 μg/ml AF 488-conjugated LPS (Molecular Probes Inc., Leiden, Netherlands) for 1 h. After the reaction was over, the cells were fixed in 3.7% paraformaldehyde, washed with PBS, and then analyzed at 494 nm/517 nm with flow cytometry as previously described 39.

### Immunofluorescence staining for formation of LPS/TLR4 complexes

To analyze the formation of LPS/TLR4 complexes on the cell surface, C2C12 cells were seeded into 4-well cell culture slides at a density of 5×10^4^ cells/ml and stabilized for 24 h and pre-treated with 20 μM tCA for 1 h and then treated with or without 1 μg/ml AF 488-conjugated LPS as previously described 40. The cells were fixed in 3.7% paraformaldehyde for 10 min, stained with anti-TLR4 antibody for 90 min at 4°C, and then incubated with AF 594-conjugated secondary antibody at RT for 1 h. After washing with PBS, the cells were also counterstained with DAPI solution for 20 min. Cell fluorescence of LPS/TLR4 complex was observed at 590 nm/617 nm using a fluorescence microscope. In addition, fluorescence intensity of DAPI-stained nuclear was recorded at 358nm/461 nm by a fluorescence microscope.

### Measurement of ROS levels

ROS was measured using 5,6-carboxy-2',7'-dichlorofluorescein diacetate (DCF-DA, Sigma-Aldrich Chemical Co.). Briefly, C2C12 cells were incubated in 6-well culture plates at a density of 3×10^5^ cells/ml for 24 h, and pre-treated with 20 μM tCA for 1 h and then incubated for 1 h in the absence or presence of 1 μg/ml LPS. The cells were stained with 10 μM DCF-DA for 15 min in the dark at 37°C. The cells were then washed with PBS and immediately recorded at 480nm/520 nm by flow cytometry (BD Biosciences, San Jose, CA, USA) as previously described 41. To compare the degree of ROS generation through fluorescence microscopic observation, the cells were stained with DCF-DA for 15 min at 37°C and then fixed with 3.7% paraformaldehyde for 10 min. The cells were washed with PBS and analyzed for ROS fluorescence intensity using a fluorescence microscope.

### Zebrafish maintenance and LPS microinjection

AB strain zebrafish, which were provided by Dr. CH Kang (Nakdong National Institute of Biological Resources, Sangju, Republic of Korea) were maintained at 28.5°C with a 14/10 h light/dark cycle according to the standard guidelines of the Animal Care and Use Committee of Jeju National University (Approval No.: 2019-0053, Jeju, Republic of Korea). Fertilized embryos were collected after natural spawning as previously described 42 and cultured in 2 mg/L methylene blue containing E3 embryo media at 28.5°C. Three days post-fertilized (dpf) zebrafish larvae were anesthetized using 0.04% tricaine (Sigma-Aldrich Chemical Co.) and LPS (0.5 mg/mL, 2 nL in each larva) was microinjected into the yolk using a Drummond NANOJECT III injector (Drummond Scientific, Broomall, PA, USA). The negative control group was injected with PBS. The larvae were washed three times after LPS microinjection and immediately placed in E3 media containing the indicated concentrations of tCA. Each group of larvae (*n* =20) was cultured at 28.5℃ for 24 h.

### NO and ROS staining in zebrafish larvae

The production of NO and ROS in zebrafish larvae was visualized using 4-amino-5-methylamino-2'7'-difluorofluorescein diacetate (DAF-FM-DA, Sigma-Aldrich Chemical Co.) and DCF-DA, respectively, 24 h after treatment as previously described 42. In brief, zebrafish embryos (4 dpf) were transferred to 24-well plates and incubated with 5 µM DAF-FM-DA and 20 µM DCF-DA for 30 min and visualized using the CELENA^®^ S Digital Imaging System (Logos Biosystems, Anyang, Gyeonggido, Republic of Korea). Fluorescence intensities were calculated using ImageJ software (Wayne Rasband, National Institute of Health, Bethesda, MD, USA) and expressed as a percentage compared to the untreated control.

### Statistical analysis

The data were analyzed with GraphPad Prism software (GraphPad Software, Inc., La Jolla, CA, USA) using one-way analysis of variance (ANOVA) for multiple comparisons, followed by Tukey's post hoc test. All numerical data are presented as the mean ± standard deviation (SD) of at least triplicate experiments. P-values of less than 0.05 were considered statistically significant.

## Results

### Effect of tCA on the proliferation of C2C12 myoblasts

**Figure 1:**
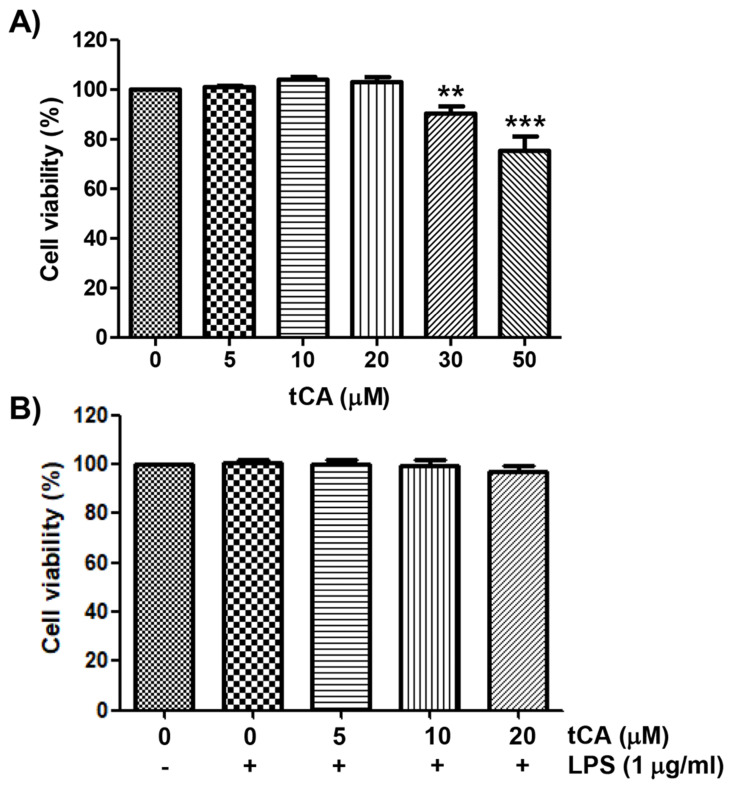
** Effect of tCA and LPS on the cell viability of C2C12 myoblasts.** Cells were treated with different concentrations of tCA alone for 24 h (A) or pre-treated with or without the indicated concentrations of tCA for 1 h before 1 mg/ml LPS stimulation for 24 h (B). Cell viability was analyzed using the MTT assay. Data indicate the mean ± SD of three independent experiments. Significant differences among the groups were determined (^**^p < 0.01 and ^***^p < 0.001, compared with the control cells).

The cytotoxic effect of tCA on C2C12 myoblasts was determined by the MTT assay. As shown in Figure 1A, at concentrations below 20 μM, tCA was not cytotoxic to C2C12 cells, but significant cytotoxicity was observed in cells treated with 30 μM or more. Subsequent experiment did not show any adverse effect on cell viability when 20 μM or less tCA was administered to 1 μg/ml LPS-treated C2C12 cells (Figure 1B).

### tCA reduces LPS-induced NO and PGE_2_ production in C2C12 myoblasts

**Figure 2:**
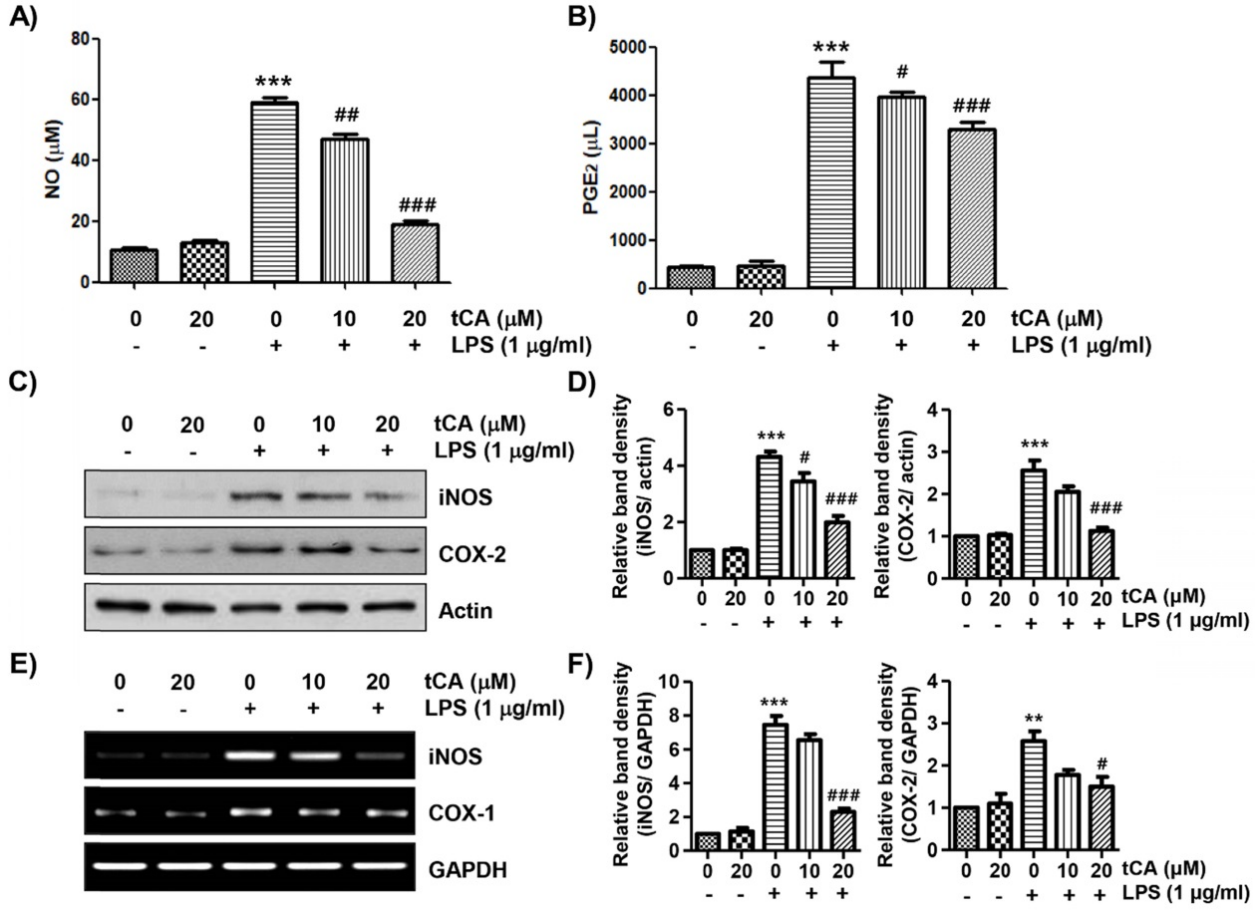
** Inhibitory effect of tCA on the production of pro-inflammatory mediators in LPS-treated C2C12 myoblasts.** Cells were treated with the indicated concentrations of tCA for 1 h and then stimulated with 1 mg/ml LPS for 24 h. (A and B) The levels of NO (A), and PGE_2_ (B) in the culture medium were determined by the Griess reaction and a commercial PGE_2_ ELISA kit. The absorbance was measured using a microplate reader. The error bars represent the SD of three independent experiments (^***^p < 0.001, *vs*. LPS-unstimulated cells; ^#^*p* < 0.05, ^##^*p* < 0.01 and ^###^*p* < 0.001, *vs*. LPS-stimulated cells). (C and E) The expression levels of iNOS and COX-2 proteins and mRNA were measured by Western blot analysis and RT-PCR, respectively. Actin and glyceraldehyde-3-phosphate dehydrogenase (GAPDH) were used as internal controls for the Western blot analysis and RT-PCR, respectively. (D and F) Relative band density. Data are expressed as the mean ± SD (^**^p < 0.01 and ^***^p < 0.001, *vs*. LPS-unstimulated cells; ^#^*p* < 0.05 and ^###^*p* < 0.001, *vs*. LPS-stimulated cells).

To evaluate the anti-inflammatory effects of tCA, the levels of inflammatory mediators such as NO and PGE_2_ in the culture supernatant were detected. As shown in Figures 2A and B, LPS stimulation markedly increased the release of NO and PGE_2_ compared to the unstimulated control, but this increase was significantly reduced in tCA-pre-treated cells in a concentration-dependent manner. Next, we investigated whether tCA could inhibit the expression of iNOS and COX-2 by LPS. According to the Western blotting and RT-PCR results, the protein and mRNA expression of iNOS and COX-2 increased by LPS was significantly suppressed in the presence of tCA (Figures 2C-F).

### tCA inhibits the production and expression of LPS-induced pro-inflammatory cytokines in C2C12 myoblasts

**Figure 3:**
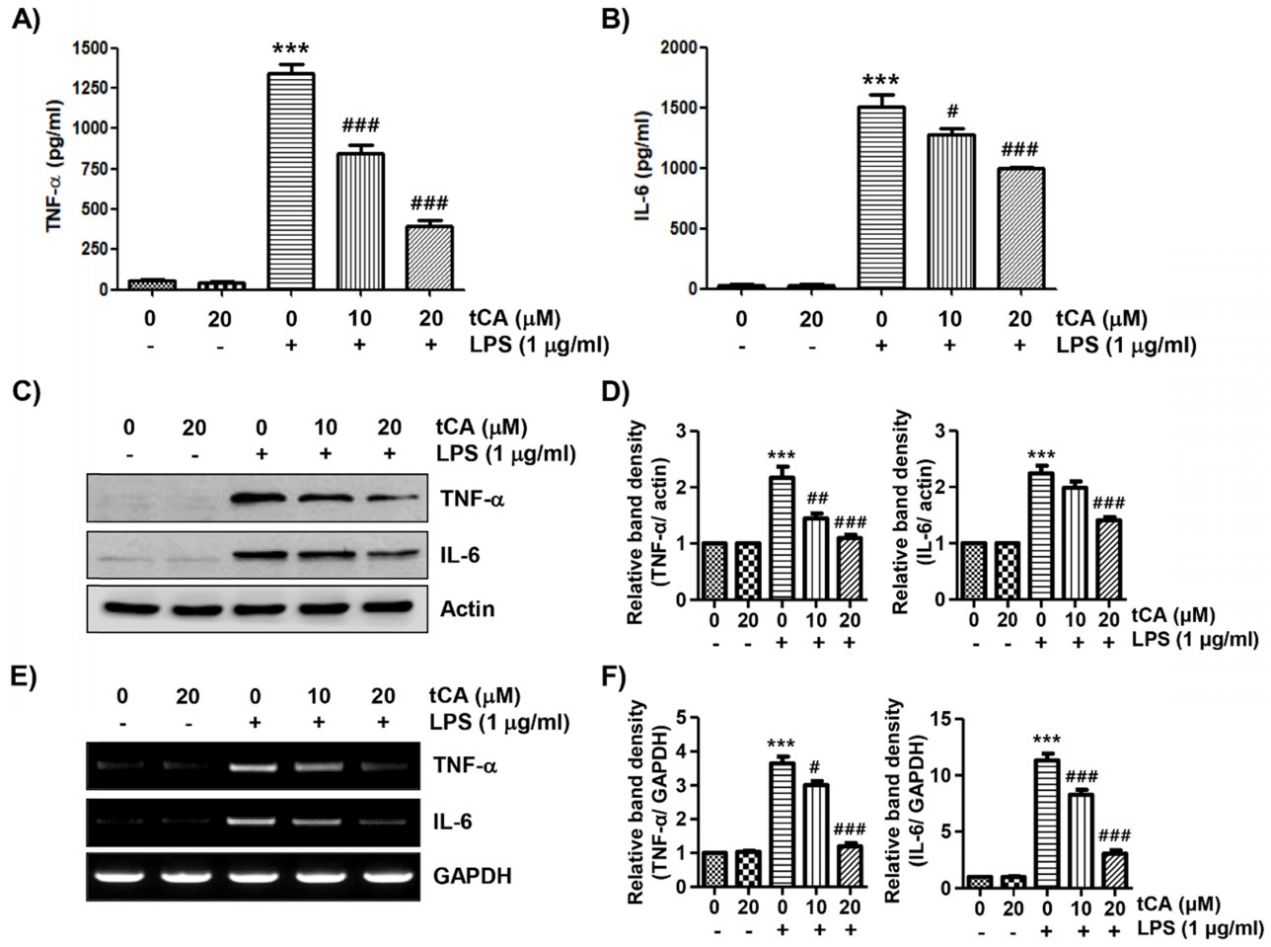
** Inhibitory effect of tCA on the production of pro-inflammatory cytokines in LPS-treated C2C12 myoblasts.** Cells were treated with the indicated concentrations of tCA for 1 h and then stimulated with 1 mg/ml LPS for 24 h. (A and B) The levels of TNF-α (A), and IL-6 (B) in the culture medium were measured using commercial ELISA kits. The absorbance was measured using a microplate reader. The error bars represent the SD of three independent experiments (^***^p < 0.001, *vs*. LPS-unstimulated cells; ^#^*p* < 0.05 and ^###^*p* < 0.001, *vs*. LPS-stimulated cells). (C and E) The expression levels of TNF-α and IL-6 proteins and mRNA were measured by Western blot analysis and RT-PCR, respectively. Actin and GAPDH were used as internal controls for the Western blot analysis and RT-PCR, respectively. (D and F) Relative band density. Data are expressed as the mean ± SD (^***^p < 0.001, *vs*. LPS-unstimulated cells; ^#^*p* < 0.05, ^##^*p* < 0.01 and ^###^*p* < 0.001, *vs*. LPS-stimulated cells).

Next, we investigated the effect of tCA on the production and expression of pro-inflammatory cytokines increased by LPS treatment. Our results showed that the amount of pro-inflammatory cytokines, including TNF-α and IL-6, released into the culture supernatant after stimulation with LPS increased significantly. However, the enhanced production of these cytokines by LPS was significantly suppressed by tCA pre-treatment, and this effect was dependent upon the tCA treatment concentration (Figures 3A and B). Subsequently, whether the inhibition of cytokine production by tCA in LPS-treated C2C12 cells was associated with the decreased expression of these genes was also investigated. As a result, LPS treatment significantly increased the expression of the two cytokine proteins, but their expression was reduced in cells pre-treated with tCA (Figures 3C-F).

### tCA suppresses the nuclear translocation of NF-κB in LPS-stimulated C2C12 myoblasts

**Figure 4:**
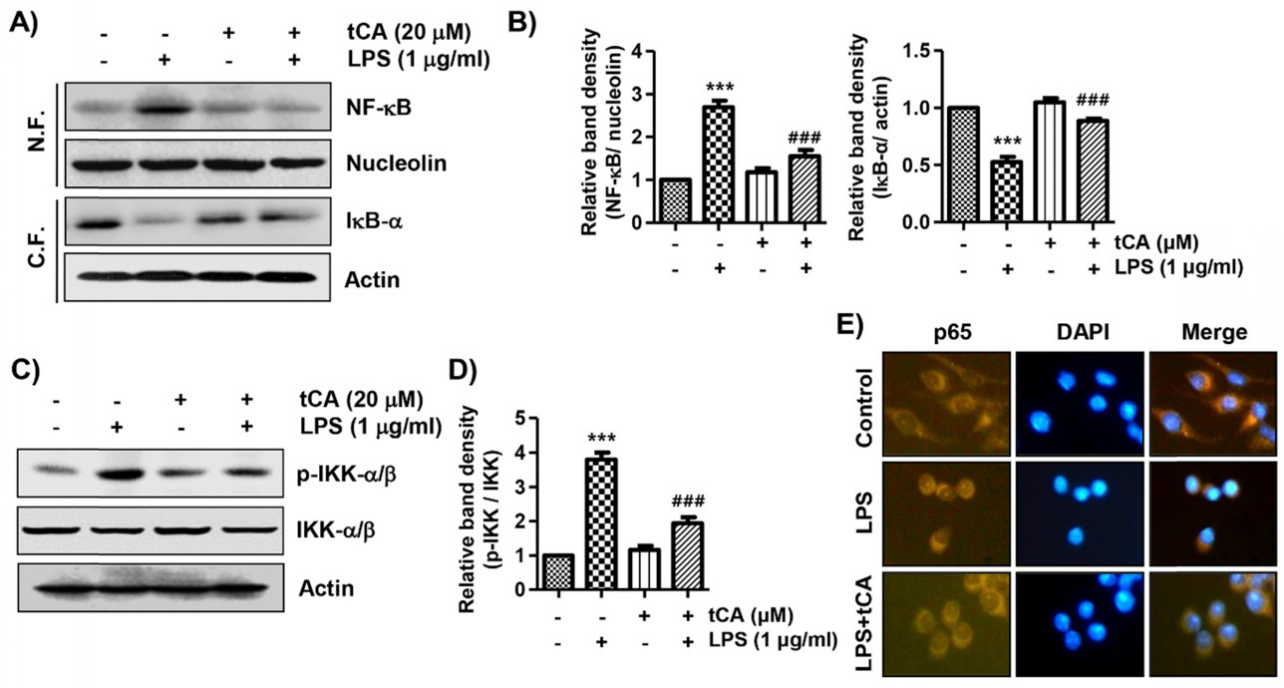
** Inhibition of LPS-induced activation of NF-κB signaling pathway by tCA in C2C12 myoblasts.** Cells were treated with 20 mM tCA alone for 24 h or pre-treated with or without 20 mM tCA for 1 h before 1 mg/ml LPS stimulation for 1 h. (A) For Western blot analysis, nuclear and cytosolic proteins were isolated, and the expression of NF-κB and IκB-a was investigated. Protein loading was confirmed by the analysis of nucleolin or actin expression in each protein extract. N.F., nuclear fraction; C.F., cytosolic fraction. (C) The expression levels of p-IKK-a/b and IKK-a/b proteins were measured by Western blot analysis using total protein. (B and D) Relative band density. Data are expressed as the mean ± SD (^***^p < 0.001, *vs*. LPS-unstimulated cells; ^###^*p* < 0.001, *vs*. LPS-stimulated cells). (E) The cells were subjected to immunofluorescence staining with NF-κB p65 antibody and representative fluorescence images were acquired using a fluorescence microscope. Brown fluorescence indicates the localization of NF-κB p65 and blue fluorescence by DAPI staining allows visualization of the nuclei.

It was further investigated whether tCA inhibits the LPS-mediated activation of NF-κB because it is a key factor controlling the transcription of pro-inflammatory mediators and cytokines. When C2C12 cells were stimulated with LPS, the expression of NF-κB in the nucleus and phosphorylated IκB kinase (p-IKK)-a/b was significantly increased compared with the control group (Figures 4A-D). By contrast, the level of IκB-α in the cytoplasm was decreased by the treatment of LPS, indicating that NF-κB was activated. However, tCA reduced the nuclear accumulation of NF-κB p65, the degradation of IκB-a and the expression of p-IKK-a/b induced by LPS. Consistent with the immunoblotting results, the increase in fluorescence intensity of NF-κB p65 observed in the nuclei of LPS-treated cells was markedly decreased by pre-treatment with tCA, as shown in Figure 4E.

### tCA diminishes the activation of the TLR4/myeloid differentiation factor 88 (MyD88) pathway induced by LPS in C2C12 myoblasts

**Figure 5:**
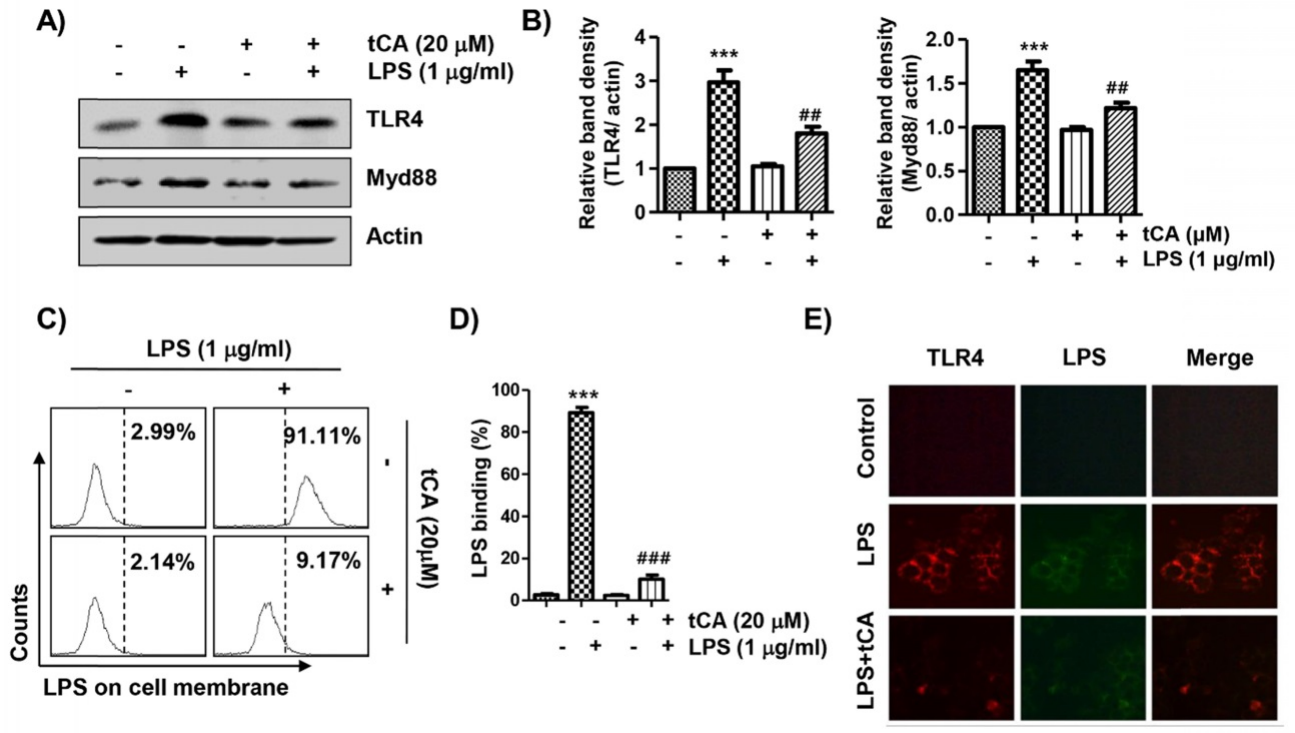
** Inhibitory effects of tCA on TLR4 and Myd88 expression and interaction between LPS and TLR4 in LPS-treated C2C12 myoblasts.** (A) Cells were pre-treated with 20 mM tCA for 1 h prior to 1 mg/ml LPS treatment for 6 h. The protein levels of TLR4 and Myd88 were determined by Western blot analysis. Actin was used as an internal control. (B) Relative band density. Data are expressed as the mean ± SD (^***^p < 0.001, *vs*. LPS-unstimulated cells; ^##^*p* < 0.01, *vs*. LPS-stimulated cells). (C-E) Cells were treated with 20 mM tCA alone for 1 h or pre-treated with or without 20 mM tCA for 1 h before 1 mg/ml AF 488-conjugated LPS stimulation for 30 min. (C and D) The level of binding of LPS to the cell surface was measured by flow cytometry. (C) The images shown represent representative plots of three replicate experiments. (D) The histogram results were statistically analyzed. Data are given as the mean ± SD of three independent experiments (^***^p < 0.001, *vs*. LPS-unstimulated cells; ^###^*p* < 0.001, *vs*. AF 488-conjugated LPS-stimulated cells). (E) The distribution of TLR4 (green) and AF 488-conjugated LPS (red) was detected by a fluorescence microscope. Experiments were repeated three times and representative fluorescence micrographs were presented.

Since LPS binds to TLR4, leading to the activation of the NF-κB signaling pathway through a Myd88-dependent and/or independent manner, we investigated the effect of tCA on the expression of TLR4 and Myd88 in LPS-treated C2C12 cells. As shown in Figures 5A and B, LPS treatment highly up-regulated the expression of TLR4 and MyD88, which was significantly reduced in the presence of tCA. In addition, it was confirmed by flow cytometry analysis that the level of AF 488-conjugated LPS bound to the cell surface was significantly reduced in tCA-pre-treated cells compared to untreated cells (Figures 5C and D). Therefore, we evaluated whether tCA could inhibit LPS binding to TLR4 in the outer membrane of C2C12 cells. The results showed that the fluorescence intensity of LPS and TLR4 was strongly observed outside the cell membrane after LPS intervention. However, their fluorescence intensity was significantly weakened in the presence of tCA (Figure 5E).

### tCA alleviates LPS-mediated generation of ROS in C2C12 myoblasts

**Figure 6:**
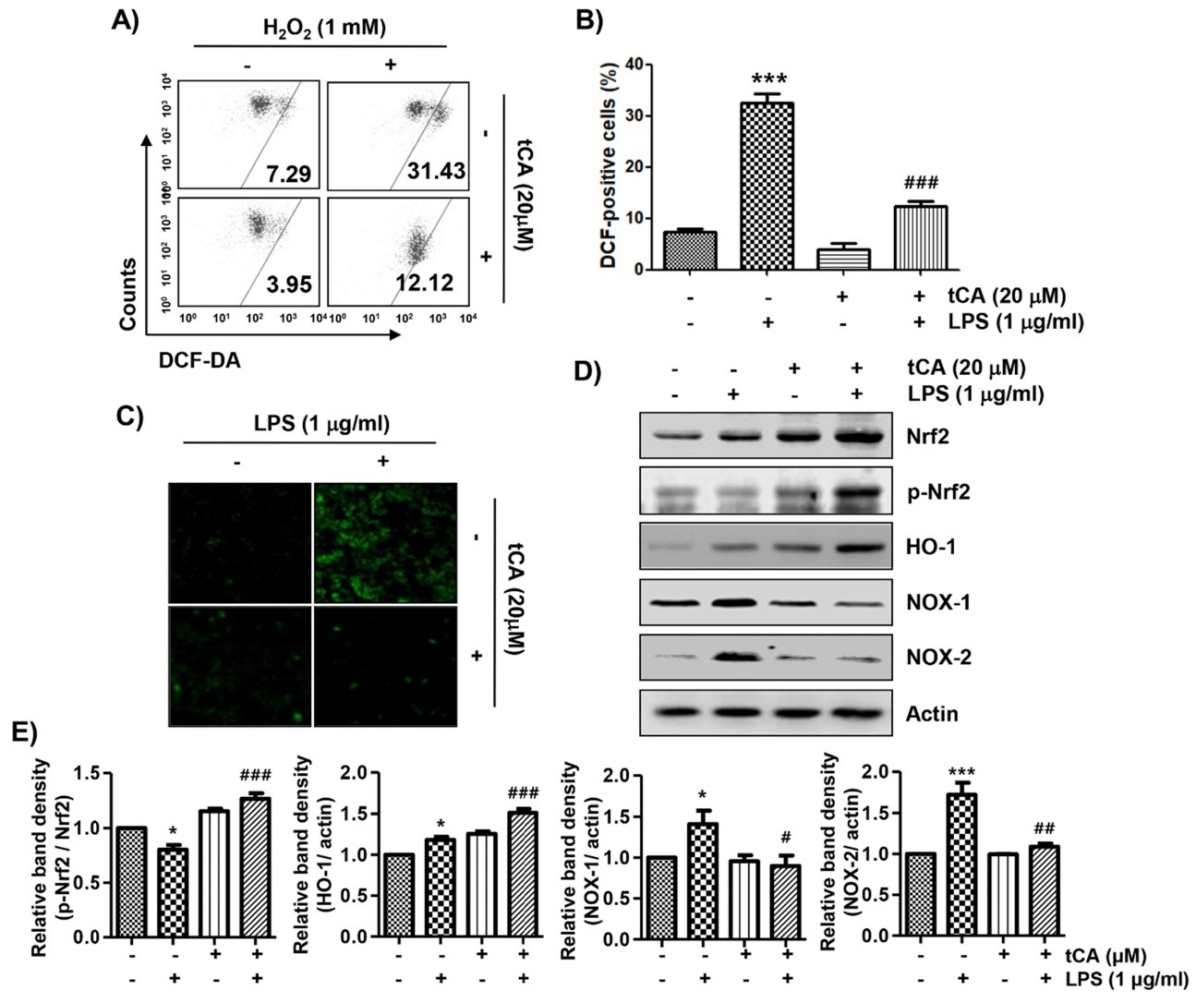
** Inhibition of ROS generation, activation of Nrf2/HO-1 signaling pathway, and suppression of NOXs expression by tCA in LPS-stimulated C2C12 myoblasts.** Cells were pre-treated with 20 mM tCA for 1 h and then treated with 1 mg/ml LPS for 1 h (A-C) or 24 h (D). (A) The DCF-DA-stained cells were collected, and then DCF fluorescence was analyzed by flow cytometry. (B) Data are given as the mean ± SD of three independent experiments (^***^p < 0.001, *vs*. LPS-unstimulated cells; ^###^*p* < 0.001, *vs*. LPS-stimulated cells). (C) ROS generation was also detected by a fluorescence microscope and representative fluorescence micrographs depicting ROS generation are presented. (D) The expression levels of Nrf2, p-Nrf2, HO-1, and NOXs proteins were measured by Western blot analysis. Protein loading was confirmed by the analysis of actin expression. (E) Relative band density. Data are expressed as the mean ± SD (^*^p < 0.05 and ^***^p < 0.001, *vs*. LPS-unstimulated cells; ^#^*p* < 0.05, ^##^*p* < 0.01 and ^###^*p* < 0.001, *vs*. LPS-stimulated cells).

Because oxidative stress also plays an important role in the inflammatory response, we investigated whether tCA could inhibit LPS-induced oxidative stress. The flow cytometry results using the DCF-DA probe showed that the increase in ROS content in C2C12 cells treated with LPS was dramatically reduced by the addition of tCA (Figures 6A and B). Consistent with the results from the flow cytometry, the increase in the fluorescence intensity of DCF-DA observed in the cells treated with LPS was also weakened by pretreatment of tCA (Figure 6C).

### tCA activates the Nrf2/HO-1 signaling pathway, but inhibits NADPH oxidases (NOXs) expression in C2C12 myoblasts

We next investigated whether the inhibitory effect of tCA on oxidative stress by LPS was related to the Nrf2/HO-1 and NOXs signaling pathways. According to the results of the Western blot analysis, the expression of Nrf2 and HO-1 was slightly increased by tCA treatment in C2C12 cells, which was associated with an increased expression of phosphorylated Nrf2 (p-Nrf2) (Figures 6D and E). However, their expression in cells co-treated with LPS and tCA was much higher than that of LPS and tCA alone treatment. On the other hand, the expression of NOX family proteins including NOX1 and NOX2, which was increased by LPS stimulation, remained at the control level in the presence of tCA.

### tCA weakens the production of NO and ROS in LPS-treated zebrafish larvae

**Figure 7:**
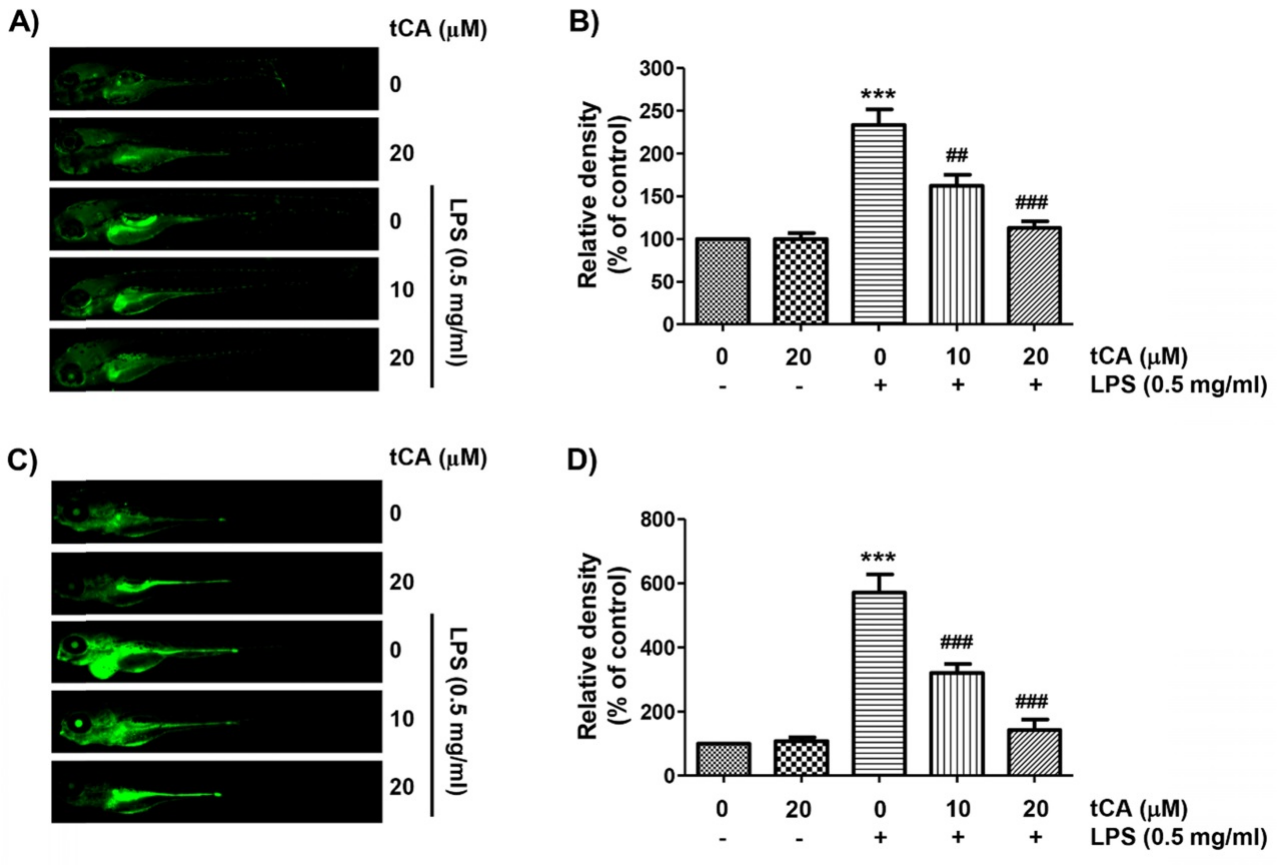
** Inhibition of LPS-induced NO and ROS generation by tCA in zebrafish larvae.** Zebrafish at 3 dpf were microinjected with 2 nL of 0.5 mg/ml LPS and placed in E3 media containing the indicated concentrations of tCA for 24 h. The larvae were incubated with 5 µM DAF-FM-DA (A and B) or 20 µM DCF-DA (C and D) for NO and ROS detection, respectively, and visualized using the CELENA® S Digital Imaging System. (B and D) Relative fluorescence intensities were calculated and expressed compared to the untreated control. Each value indicates the mean ± SD and is representative of three independent experiments with 10 fish for each group. Significant differences among the groups were determined (^***^p < 0.001, *vs*. LPS-unstimulated larvae; ^##^*p* < 0.01 and ^###^*p* < 0.001, *vs*. LPS-stimulated larvae).

As tCA downregulates inflammatory and oxidative responses in C2C12 cells, we wondered if tCA had a similar effect in the *in vivo* model and demonstrated it using the zebrafish model. According to the results of DAF-FM-DA staining, LPS microinjection significantly increased NO generation. However, in the presence of tCA within a non-toxic range, the LPS-induced NO generation gradually decreased in a concentration-dependent manner (Figures 7A and B). In addition, we confirmed by DCF-DA staining that the increased ROS accumulation in LPS-microinjected zebrafish larvae was dose-dependently abrogated in the presence of tCA (Figures 7C and D).

## Discussion

To evaluate the anti-inflammatory efficacy of tCA, we first investigated the effect of tCA on the production of NO and PGE_2_. Among them, NO is synthesized from L-arginine by NO synthase, and acts as not only a potent activator but also an inhibitor of the inflammation 43-47. Several studies suggested that NO plays a critical role in normal physiological conditions such as neurotransmission, vasodilation, and immune defense 43,44. Lee et al. demonstrated that NO suppresses LPS-induced inflammation in a mouse asthma model by attenuating the interaction of IKK and heat shock protein 90 45. In addition, Raychaudhuri et al. reported that NO blocks LPS-stimulated NF-κB activation in alveolar macrophages 46. Although the several studies suggested that NO plays a potential inhibitor of the inflammation, the role is restricted in almost bronchial system. In this regard, the role as pro-inflammatory mediator of NO is still superiority. Excessive NO formation due to increased iNOS expression promotes the inflammatory response and increases oxidative stress and tissue damage 47. COX enzymes catalyze the conversion of arachidonic acid to prostaglandins, including PGE_2_, a group of hormone-like substances that participate in various body functions 8,7. However, excessive PGE_2_ production, promoted by the increased activity of COX-2 following various inflammatory stimuli, also plays an important role as an inflammatory mediator 43,44. Therefore, inhibitors of the excessive production of these inflammatory mediators can be regarded as therapeutic agents against inflammation-related diseases. Our data indicated that the up-graduated secretion of NO and PGE_2_ in LPS-stimulated C2C12 myoblasts was progressively inhibited at increasing concentrations of tCA, which was associated with inhibition of the expression of iNOS and COX-2 protein and mRNA. These data demonstrated that the anti-inflammatory effect of tCA was at least due to the reduced expression of iNOS and COX-2, which are involved in NO and PGE_2_ production, and support the results of previous studies observed in human knee articular chondrocytes, and murine microglial cells 22,24,27,30. Furthermore, the present finding from C2C12 cells that tCA suppressed NO production following by LPS exposure, is accords with the result from zebrafish.

During the inflammatory response, macrophages secrete multiple pro-inflammatory cytokines that are involved in various signaling pathways producing autocrine and/or paracrine effects 48,49. All of these are essential components for the initiation and improvement of the inflammatory response, and their expression is also increased by the LPS stimulation of muscle cells 3,4,50. Moreover, they can accelerate the inflammatory response by activating or increasing the expression of pro-inflammatory mediators as well as other pro-inflammatory cytokines 4,21. Therefore, the level of pro-inflammatory cytokines has been applied as an indicator to evaluate anti-inflammatory efficacy in muscle cells. In the current study, we found that tCA reduced the production of TNF-α and IL-6 in LPS-stimulated C2C12 myoblasts by suppressing their expression. Consistent with our results, Kim et al. 51 also reported similar effects in RAW 264.7 macrophages stimulated with LPS, and these results well support the anti-inflammatory efficacy of tCA found in several experimental models 31,33,52.

Among various intracellular signaling pathways, NF-κB has been identified as the most important transcription factor in regulating the expression of pro-inflammatory factors by LPS stimulation 53,54. Typically, NF-κB forms a complex with the inhibitory subunit IκB-α and remains inactive in the cytoplasm. When IκB-α is phosphorylated by IKK-α/b and degraded through the ubiquitin-proteasomal pathway, NF-κB migrates to the nucleus, triggering transcriptional activation of inflammation-inducing genes and catabolic enzymes. As is well known, TLR4, a pathogen pattern receptor on the cell surface, can recruit MyD88 when bound to LPS to induce NF-κB activation 55,56. TLR4/Myd88/NF-κB is known as a classical signaling pathway whose activation is consider to be responsible for the massive inflammation and considered to be a valuable and promising therapeutic target against inflammation related diseases 57-60. Yu et al. 58 reported that *Panax quinquefolius L*. Saponins protect myocardial ischemia reperfusion through blocking TLR4/Myd88/NF-κB signaling pathway suggesting it can be potential therapeutic target pathway for preventing myocardial ischemia. More recently, Xu et al. demonstrated that metformin, a natural compound found in the plant *Galega officinalis*, suppress LPS-induced inflammatory response via blocking TLR4/ NF-κB signaling pathway in bovine mammary epithelial cells 60. Therefore, to elucidate the mechanism of inhibition of the LPS-induced inflammatory response by tCA, the expression levels of NF-κB, IκB-α, IKK-α/b, TLR4, and MyD88 were investigated. Our results showed that in LPS-stimulated C2C12 cells, tCA effectively reversed the translocation of NF-κB from the cytoplasm to the nucleus, degradation of IκB-α, and phosphorylation of IKK-α/b, an essential step for NF-κB activation. Consequently, it appears that the NF-κB pathway participated in the anti-inflammatory process of tCA, and these results are in good agreement with the anti-inflammatory mechanisms of this compound observed in several previous studies 28-31. Furthermore, tCA suppressed LPS-induced expression of TLR4 and MyD88 proteins, and the level of LPS binding on the cell surface was significantly reduced in the cells pre-treated with tCA compared to untreated cells. These results suggest that tCA can inhibit LPS-mediated inflammatory action in C2C12 myoblasts by repressing TLR4-mediated NF-κB signaling through an antagonistic effect on the binding of LPS and TLR4.

Meanwhile, endogenous free radicals like ROS play an important role in host defense. However, excess ROS can cause oxidative damage to cellular macromolecules, and has been shown to play a key role in initiating and promoting inflammation-related diseases by upregulating the production of inflammatory mediators and cytokines 10,11. According to our results, tCA strongly inhibited LPS-induced ROS formation in C2C12 myoblasts and in zebrafish, supporting that the use of tCA as an antioxidant for the management of oxidative stress associated with inflammatory responses. HO-1, a typical cytoprotective and inducible enzyme, is one of the downstream anti-oxidative phase II enzymes dependent upon transcription factor Nrf2. For Nrf-2 to act as a transcription factor, it must be liberated from Kelch-like ECH-associated protein 1, a negative regulator of Nrf2, in the cytoplasm and phosphorylated before translocation to the nucleus 61,62. Unlike HO-1, NOXs are key enzymes involved in the production of ROS as important regulators of redox homeostasis 63,64. NOX consists of seven members, of which NOX2 acts as a major contributor of ROS generation during skeletal muscle contraction 65,66. And NOX1, which is highly expressed in skeletal muscle along with NOX2 and NOX4 65,67, is increased by myostatin in C2C12 myoblasts 68, but its physiological role in regulating ROS production has not been well identified. In addition, the accumulated evidence supports that the activation of NOX1 and NOX2 can participate in LPS-induced inflammatory responses as well as ROS production 15,69,70. Therefore, we investigated the effect of tCA on the protein levels of Nrf2 and HO-1 in LPS-stimulated C2C12 myoblasts, and found that tCA increased their levels in the presence of LPS. Additionally, the degree of phosphorylation of p-Nrf-2 was also markedly increased in cells co-treated with tCA and LPS compared to the group treated with tCA or LPS alone, indicating that the activity of Nrf-2 as a transcription factor was improved. However, pre-treatment with tCA inhibited the expression levels of NOX1 and NOX2 in LPS-stimulated C2C12 myoblasts. These observations provide the possibility that tCA may be responsible for the anti-inflammatory and antioxidant effects in C2C12 myoblasts stimulated with LPS through mediating the Nrf2/HO-1 axis and the NOXs signaling pathway. However, the relationship between Nrf-2 and the NOX signaling system and the role of their upstream regulators should be further investigated in the future. Moreover, further studies using HO-1 siRNA are needed to identify the role of tCA on the down-regulation of TLR4/NF-κB signaling pathway in LPS-stimulated C2C12 myoblasts. Since *in vivo* experiments can provide a better understanding of efficacy assessment at the organism level, the anti-inflammatory and antioxidant potential of tCA identified in C2C12 myoblasts was further confirmed in the zebrafish model. The present results showed the ability of tCA to inhibit inflammatory and oxidative reactions in the LPS-microinjected zebrafish larvae model by reducing NO and ROS generation. Although these results support our *in vitro* results, additional mechanistic studies are needed to interpret the mechanisms related to the anti-inflammatory and antioxidant efficacy of tCA in *in viv*o model.

**Figure 8:**
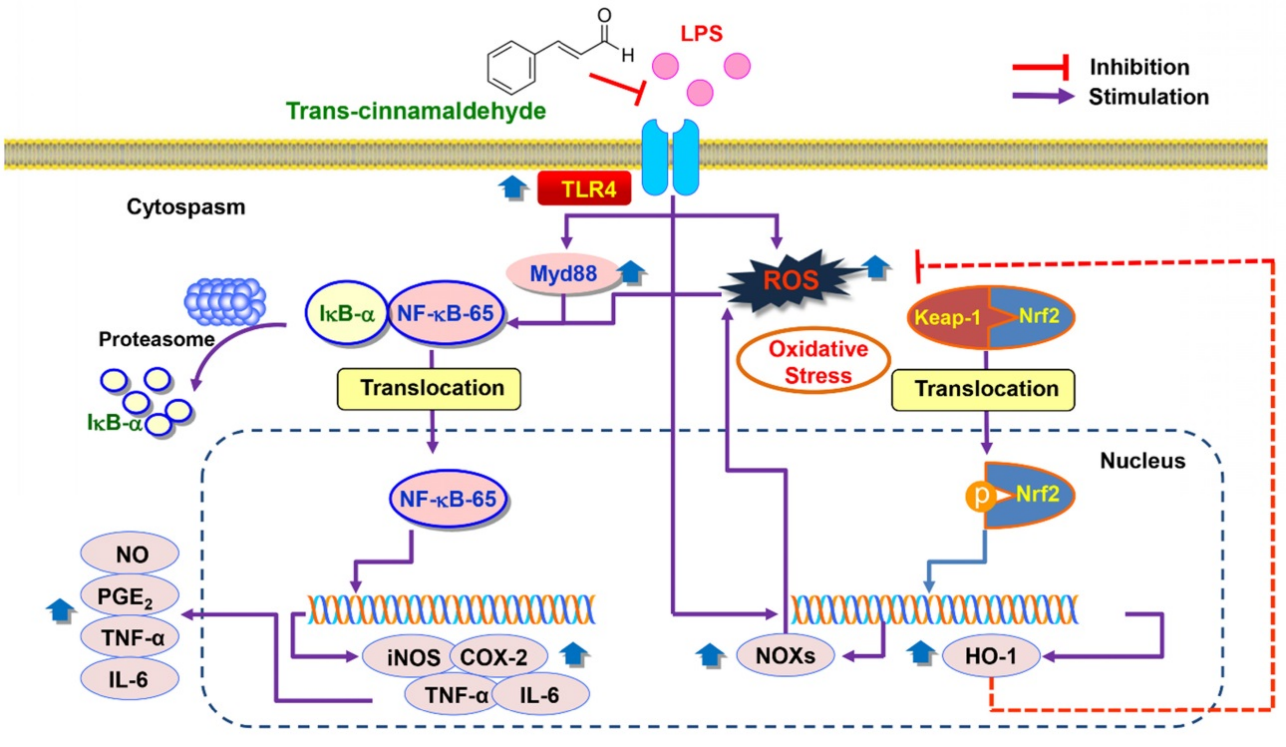
Proposed mechanism of anti-inflammatory and antioxidant effects of tCA in C2C12 myoblasts.

In summary, the current study showed that pro-inflammatory enzymes such as iNOS and COX-2 and cytokines including TNF-α and IL-6 were downregulated by tCA due to inhibition of the TLR4/NF-κB signaling pathway in LPS-stimulated C2C12 myoblasts. tCA also attenuated the accumulation of ROS, which was associated with the activation of the Nrf2/HO-1 signaling pathway and inhibition of NOXs proteins expression (Figure 8). In addition, Furthermore, the anti-inflammatory and antioxidant activities of tCA were also confirmed in an *in vivo* zebrafish model. Building on these results, tCA will provide a pharmacological basis for exciting future research in preventing and reducing the risk of various inflammation and oxidative stress-mediated muscle diseases.

## Figures and Tables

**Table 1:** Primer information for RT-RCR.

Gene	Forward sequence	Reverse sequence
COX-2	GCGACATACTCAAGCAGGAGCA	AGTGGTAACCGCTCAGGTGTTG
iNOS	GAGACAGGGAAGTCTGAAGCAC	CCAGCAGTAGTTGCTCCTCTTC
IL-6	TACCACTTCACAAGTCGGAGGC	CTGCAAGTGCATCATCGTTGTTC
TNF-α	GGTGCCTATGTCTCAGCCTCTT	GCCATAGAACTGATGAGAGGGAG
GAPDH	CATCACTGCCACCCAGAAGACTG	ATGCCAGTGAGCTTCCCGTTCA
